# DNA Manipulation and Single-Molecule Imaging

**DOI:** 10.3390/molecules26041050

**Published:** 2021-02-17

**Authors:** Shunsuke Takahashi, Masahiko Oshige, Shinji Katsura

**Affiliations:** 1 Division of Life Science and Engineering, School of Science and Engineering, Tokyo Denki University, Hatoyama-cho, Hiki-gun, Saitama 350-0394, Japan; stakahashi@mail.dendai.ac.jp; 2 Department of Environmental Engineering Science, Graduate School of Science and Technology, Gunma University, Kiryu, Gunma 376-8515, Japan; oshige@gunma-u.ac.jp; 3 Gunma University Center for Food Science and Wellness (GUCFW), Maebashi, Gunma 371-8510, Japan

**Keywords:** DNA, single-molecule, DNA manipulation, fluorescence microscope

## Abstract

DNA replication, repair, and recombination in the cell play a significant role in the regulation of the inheritance, maintenance, and transfer of genetic information. To elucidate the biomolecular mechanism in the cell, some molecular models of DNA replication, repair, and recombination have been proposed. These biological studies have been conducted using bulk assays, such as gel electrophoresis. Because in bulk assays, several millions of biomolecules are subjected to analysis, the results of the biological analysis only reveal the average behavior of a large number of biomolecules. Therefore, revealing the elementary biological processes of a protein acting on DNA (e.g., the binding of protein to DNA, DNA synthesis, the pause of DNA synthesis, and the release of protein from DNA) is difficult. Single-molecule imaging allows the analysis of the dynamic behaviors of individual biomolecules that are hidden during bulk experiments. Thus, the methods for single-molecule imaging have provided new insights into almost all of the aspects of the elementary processes of DNA replication, repair, and recombination. However, in an aqueous solution, DNA molecules are in a randomly coiled state. Thus, the manipulation of the physical form of the single DNA molecules is important. In this review, we provide an overview of the unique studies on DNA manipulation and single-molecule imaging to analyze the dynamic interaction between DNA and protein.

## 1. Introduction

Single-molecule imaging and DNA manipulation provide new analytical methods in molecular biology and biochemistry [1,2,3,4,5]. The advancements in microscope and fluorescence imaging technology for biomolecules enabled the direct observation of DNA and proteins at the single-molecule level. Unlike electron microscopy (EM) [6] and atomic force microscopy (AFM) [7], fluorescence single-molecule imaging technologies allow direct observation of fluorescently labeled biomolecules in an aqueous solution. This makes the determination of the dynamic behavior of individual biomolecules possible [2,3]. Information obtained from single-molecule imaging and DNA manipulation contains not only the average behavior of biomolecules but also the distribution of the dynamic behavior of individual biomolecules. For the past two decades, single-molecule imaging and DNA manipulation have provided insights into biological processes based on the dynamic behavior of individual biomolecules. Here, we describe the features and measurements of single-molecule imaging and DNA manipulation.

### 1.1. Statistical Average in Biological Fields

In a conventional bulk assay, several millions of biomolecules are submitted for analysis. As a result, the bulk assay provides only the average behavior of molecules. The summation of the behaviors of a large number of molecules converges on a Gaussian distribution due to the central limit theorem in basic statistics, despite the difference between the source distribution of individual molecules and Gaussian distribution. As an example, two model distributions are assumed, whose averages and standard deviations are completely equal. Even if the original distributions, which show the behavior of the individual samples, are completely different, the summation generates quite similar distributions. Thus, distinguishing between the two Gaussian distributions is difficult. This is because the summation of individual distributions of 100 samples corresponds to the average of the behaviors of 100 molecules. Thus, conventional bulk assays are conducted to provide information on only the average behavior of a large number of molecules, but not on the distribution of individual molecules.

### 1.2. Flexibility of DNA Molecule as a Polymer

The DNA molecule is a long-chain polymer that consists of two antiparallel polynucleotide chains that coil around each other to form a double helix [8,9]. Non-B-DNA structures, such as A-form or Z-form, are induced by a high salt or high ethanol concentration [10,11,12], whereas the double helix of DNA in an aqueous solution of low ion concentration forms a B-DNA structure. Both the physical form and positions of the individual DNA molecules are easily changed by external forces in an aqueous solution [13,14,15]. However, the physical form of the DNA molecules fluctuates in the absence of external forces due to the Brownian motion derived from the flexibility of the DNA molecules [16,17,18]. This is because the DNA molecules tend to increase the variety of physical forms due to the increase in entropy. In particular, DNA molecules in an aqueous solution exhibit a right-handed double-helical B form structure [19]. Conversely, circular DNA and terminal immobilized linear DNA molecules induce a supercoiled state. In particular, high negative superhelicity locally induces non-B DNA structures, such as interior loops, cruciforms, and Z-forms [20,21,22]. Essentially, the energy necessary for DNA supercoiling is almost of the same order as the thermal energy at room temperature. Local DNA supercoiling with various densities can be easily induced by thermal agitation at room temperature in conventional bulk assays [23]. This indicates that understanding the physiological role of the structure of DNA molecules in biological processes, such as DNA replication, repair, and recombination, is extremely difficult; even the supercoiled state of DNA is controlled in advance. Therefore, it is important to control the physical form of DNA molecules in situ.

### 1.3. Flexibility of Genomic DNA Molecules

**Figure 1:**
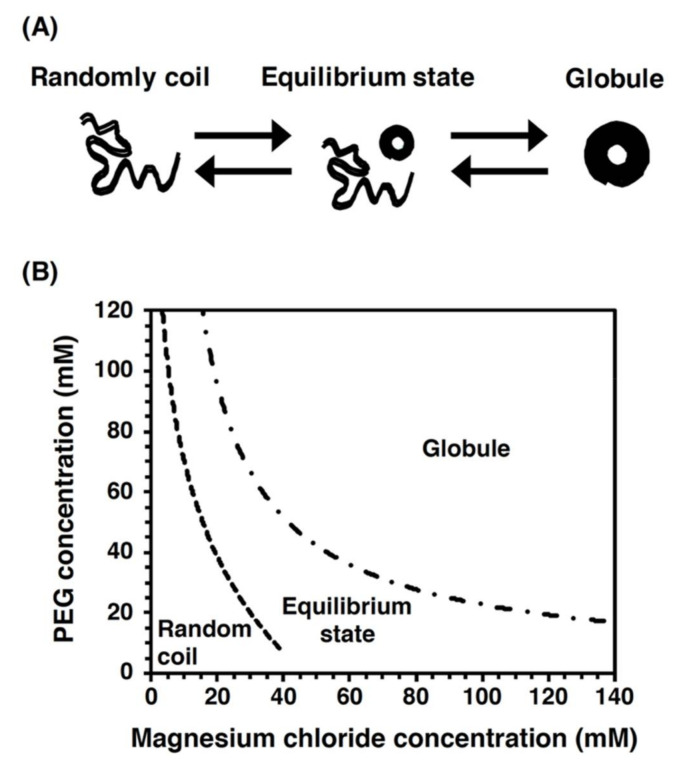
Outline of reversible phase transition between the randomly coiled state and globular state of the DNA molecules. (**A**) The phase transition of the physical form of DNA molecule using condensing agents. By adding condensing agents, the DNA molecule is transformed from a randomly coiled structure to a globular structure. By removing condensing agents, the globular DNA molecule reverts to the randomly coiled structure. The phase transition between the globular and the randomly coiled structures can be reversibly induced in a DNA molecule. (**B**) Relationship of PEG and salt concentrations with the phase transition between the globular and the randomly coiled structures. The dashed line denotes the phase transition between the randomly coiled structure and the equilibrium states, which involve the globular and the randomly coiled structures. The dot chain line denotes the phase transition between the globular structure and the equilibrium states, which involve the globular and the randomly coiled structures.

Long-chain DNA molecules over 100 kb are easily broken up by shear stress, along with flow and vortex. Thus, the size of the DNA molecules that can be handled in an aqueous solution is limited to 100 kb. However, in the fields of life science, such as genomic engineering and synthetic biology, it is important to handle genomic DNA without fragmentation (e.g., in the artificial synthesis of genomic DNA and transfection or transformation of synthesized long-chain DNA into cells) [24,25,26,27,28,29]. Genomic DNA molecules can be chemically aggregated using condensing agents, such as polyethylene glycol (PEG) and/or low-molecular-weight inorganic salts (Figure 1A) [30,31,32]. Globular DNA molecules can be prepared via phase transition from a randomly coiled structure to a globular structure (Figure 1B) [33,34]. Because the globular DNA molecule is protected from physical stress, it can be manipulated without fragmentation induced by shear stress [35,36]. The reversible transition of the globular structure of the DNA molecules to the randomly coiled structure is performed by removing the PEG and/or salt [37].

## 2. DNA Manipulation

The conformation of DNA molecules in an aqueous solution flexibly fluctuates due to Brownian motion (see Section 1.2). The DNA molecules behave as a wormlike chain, and voluntarily shorten the distance between the ends as their entropies increase. Thus, fluorescently stained DNA molecules appear as bright spots under a fluorescence microscopic field. Therefore, measuring the full length of the single DNA molecules under the relaxed state is difficult. Even though bright spots are detected, it is difficult to determine the contour lengths between the bright spots of the protein bound to DNA and the DNA terminus in randomly coiled DNA molecules. For this reason, the manipulation of the physical form of the DNA molecules is necessary for obtaining information on the binding sites of proteins. 

Here, we introduce several methods for single DNA molecule manipulation that have been developed: (1) DNA manipulation by orientation forces, (2) DNA manipulation by optical tweezers, (3) the DNA manipulation of terminal immobilized DNA by fluid flow or electric fields, (4) DNA manipulation by magnetic tweezers, (5) DNA manipulation by interface movement, (6) DNA origami, and (7) nanopores and DNA manipulation.

### 2.1. DNA Manipulation by Orientation Forces

**Figure 2:**
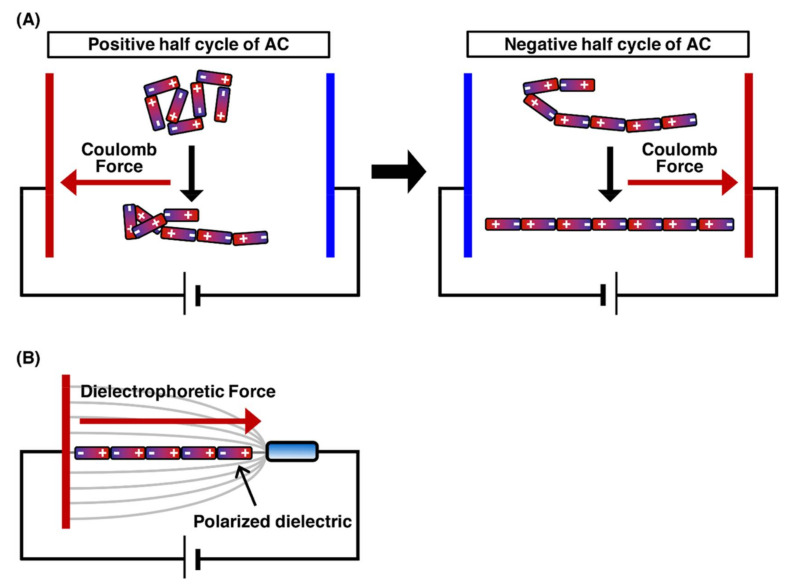
Outline of the stretching of the DNA molecule by orientation manipulation. The charge is induced on the single DNA molecule by the electric field. The Coulomb forces are exerted on the induced charges, and then, the DNA molecule is stretched by the Coulomb forces. (**A**) The stretching process for DNA molecule by the application of AC electric field. (**B**) The stretching of DNA molecule by orientational force.

The application of an electric field to linear DNA molecules results in the local induction of charges opposite to the closer electrode on the DNA molecules. Figure 2 presents the orientation force exerted on DNA molecules. Each end of the charged DNA molecules is attracted by the Coulomb force toward the near electrode, which results in the stretching of the DNA molecules toward each electrode [38,39]. However, when a direct current (DC) electric field is applied, fluid flow is generated by bubbles with the electrode reactions, thus preventing the manipulation of DNA molecules. Conversely, when an alternating current (AC) electric field with cycling for the positive half-cycle and negative half-cycle is applied, the fluid flow generation by bubbles with the electrode reactions is significantly suppressed. This is because the electrode reactions generated during the positive half-cycle can be canceled by those generated during the negative half-cycle. Therefore, the application of an AC electric field is suitable for DNA manipulation by orientation forces (Figure 2A) [40,41]. The dielectrophoretic force (the interaction between an induced dipole on the molecule and a nonuniform electric field) is generated according to the dielectric properties of the molecule and medium. In a positive dielectrophoretic force, the molecule, which is more polarizable than the medium, is attracted in a direction toward the region of high electric field gradients. On the other hand, in a negative dielectrophoretic force, the molecule, which is less polarizable than the medium, is attracted in a direction away from the region of high electric field gradients. By controlling the dielectrophoresis force, single DNA molecules were manipulated by the application of an AC electric field with a frequency of 1 MHz at a field strength of 1 MV/m (Figure 2B) [42]. The application of an AC electric field enables the efficient alignment of hundreds of individual DNA molecules under fluorescence microscopic fields. The field strength required for DNA manipulation, i.e., 1 MV/m, is quite high; therefore, electrode miniaturization is necessary to generate a sufficient electric field. Electrode miniaturization also suppresses heat generation by Joule heating, thus hindering the manipulation of DNA molecules. For this reason, it should be noted that a higher salt concentration causes significant heat generation and electrode reactions. For DNA manipulation by the electric fields, it is important to keep the solution under a low ionic strength condition in the concentration range 1–100 µM for multivalent cations, such as calcium ions, magnesium ions, zinc ions and aluminum ions, that reduce polarization by binding to the DNA backbone [40].

### 2.2. DNA Manipulation by Optical Tweezers

**Figure 3:**
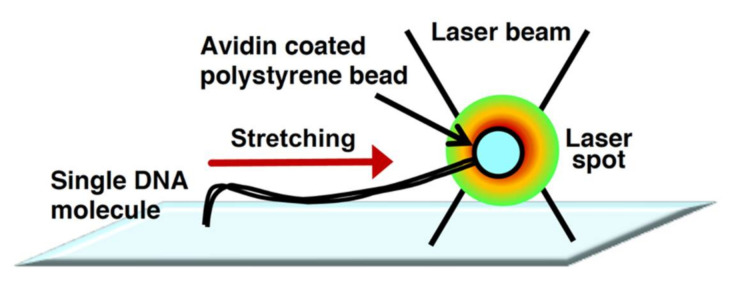
Physical manipulation of a single DNA molecule attached to microbeads using optical tweezers. Conceptual diagram of DNA manipulation by optical tweezers.

The physical principles of laser trapping were discovered by Arthur Ashkin in 1970 [43,44]. In laser trapping, an extremely bright and squeezed laser beam is refracted by a particle at the focal point. The photons that have momentum exert a force on the microparticle during refraction. Due to this power, particles are trapped without physical contact around the focal point of the laser beam. The manipulation technology for the particle position is called optical tweezers [45,46]. However, the manipulation of randomly coiled DNA molecules by optical tweezers is extremely difficult. Thus, by attachment to a particle at one end of the linear DNA molecule, the single DNA molecule is indirectly manipulated by the trapped particle by optical tweezers. The indirectly manipulated single DNA molecules can be stretched by the fluid flow or an electric field [47,48,49]. However, the fluid flow cannot uniformly stretch the trapped single DNA molecules. To enable the uniform stretching of single DNA molecules, they need to be physically stretched from one end to the other end using particles via laser trapping. Figure 3 demonstrates that one end of a single DNA molecule is immobilized on the surface, whereas the other end is attached to a particle. The manipulation of the particle under this condition enables the uniform stretching of the trapped single DNA molecules [50,51]. The use of dual-beam optical tweezers can successfully stretch DNA molecules whose termini are attached to different particles and control their tension based on the particle position [52,53]. The development of optical tweezers has made it possible to determine the force in the order of piconewtons to nanonewtons, which facilitates progressive analyses of the dynamic interaction between DNA and protein at a single-molecule level [54].

### 2.3. Manipulation of Genomic DNA by Laser Trapping

**Figure 4:**
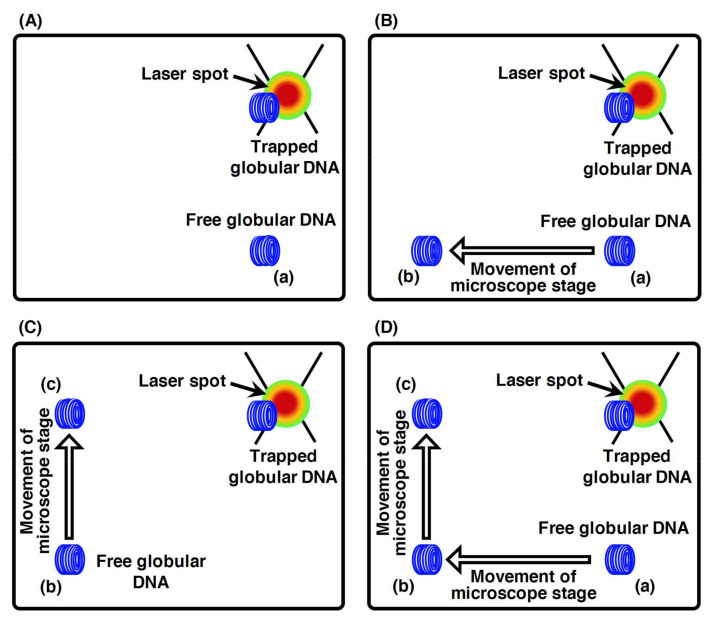
Direct laser trapping of the globular DNA molecule. Schematic illustration of the changes in the positions of free and trapped globular DNA molecules induced by the movement of the microscope stage. (**A**) A globular DNA molecule was trapped by laser. (**B**) When the microscope stage moved to the left, the free globular DNA molecule moved to the left position from (a) to (b) with the movement of the microscope stage. On the other hand, the trapped globular DNA molecule stayed at the upper right position of the microscope stage. (**C**) When the microscope stage moved up, the free globular DNA molecule moved to the upper position from (b) to (c) with the movement of the microscope stage. On the other hand, the trapped globular DNA molecules stayed at the upper right position of the microscope stage. (**D**) Merging of steps A, B and C. Refer to [55] for details.

As described in Section 2.2, the application of laser trapping causes the particles to be spatially trapped without physical contact at the focal position of the laser beam. However, the trapping of randomly coiled DNA molecules in an aqueous solution is extremely difficult. This is because there is no significant difference in the refractive index between water molecules and low-density randomly coiled DNA molecules in the solvent. However, high-density globular DNA can be optically trapped using a neodymium-doped yttrium aluminum garnet (Nd:YAG) laser at a wavelength of 1064 nm. Figure 4 presents the trapping of globular DNA by a laser beam [55]. As can be seen from the figure, the trapped globular DNA is not moved simultaneously with the microscope stage, whereas the nontrapped globular DNA is moved simultaneously with the microscope stage (Figure 4A–D). Thus, globular DNA manipulation can be achieved by laser trapping.

### 2.4. DNA Manipulation by Electric Field and Fluid Flow

**Figure 5:**
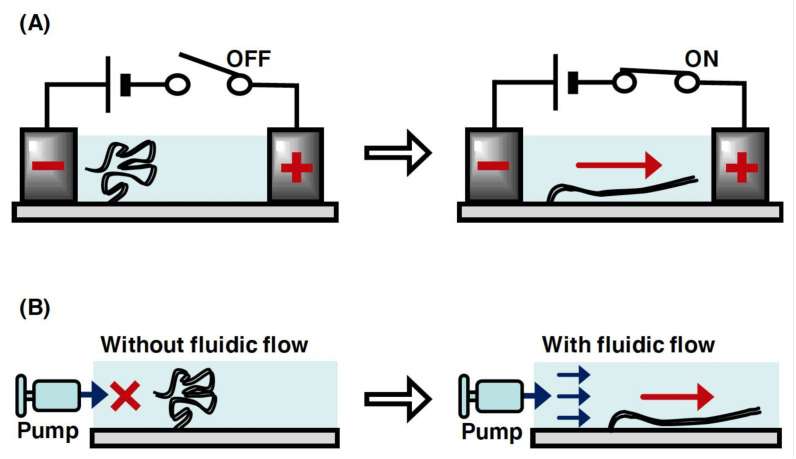
Conceptual diagram of the manipulation of single DNA molecules with one end immobilized on a surface. (**A**) Stretching manipulation of single DNA molecules by a DC electric field. The DNA molecule is relaxed without the application of electric field but stretched with the application of it. (**B**) Stretching of single DNA molecule by the fluid flow. The DNA molecule is relaxed without the fluid flow but stretched with the fluid flow.

In a microchannel device, individual linear DNA molecules with one end immobilized on a glass surface can be stretched by the fluid flow or an electric field generated using miniaturized electrodes (Figure 5A,B). The application of a DC electric field at 10 V/cm enabled the stretching of single DNA molecules without a fluid flow in a microchannel [56,57]. However, the single DNA molecules were not stretched without both the fluid flow and DC electric field, which resulted in the randomly coiled state [57]. This indicates that the negative charge of the phosphate backbone of DNA strands was attracted toward the anode upon applying a DC electric field. In terms of the fluid flow, the individual linear DNA molecules can be stretched by manipulating the fluid flow using a microsyringe pump in a microchannel (Figure 5B) [58]. This is because the fluid flow generates an external force to transform the physical form of the single DNA molecules from the randomly coiled state to the stretched state. The solution can be controlled at the reaction field by exchanging with the solution that is newly injected into the microchannel device. Upon applying the fluid flow and electric field, several dozens of stretched single DNA molecules can be observed under the same fluorescence microscopic fields. This enables an efficient analysis of the dynamic interaction between DNA and protein in a high-throughput manner. In particular, by using a simple micro- or nanofabricated glass surface with lipid bilayer coating, a single-molecule technique, called DNA curtains, has been developed to align the arbitrary patterns of thousands of single DNA molecules in a microchannel [59,60,61,62,63]. This technique has provided a powerful experimental platform for the concurrent observation of hundreds or thousands of dynamic interactions between DNA and protein [5]. 

Contrary to the method using optical tweezers (see Section 2.2), stretching methods based on fluid flow or electric fields do not provide uniform stretching of the DNA molecules. This is because the local force for the stretching of the linear DNA molecules is approximately proportional to the contour length from the corresponding point to the free end of the DNA molecule. Thus, the stretching force around the free end region of the linear DNA molecule is much less than that around the immobilized end region of the DNA molecule.

### 2.5. DNA Manipulation by Magnetic Tweezers

**Figure 6:**
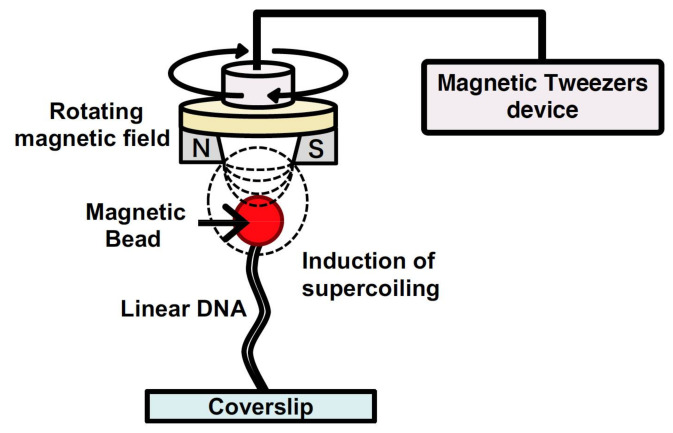
Induction of supercoiling in the single DNA molecules by magnetic tweezers.

Magnetic tweezers have been utilized to manipulate single DNA molecules and to conduct analysis of the mechanical properties of the interaction between DNA and protein at a single-molecule level [64,65]. A single DNA molecule is tethered to a surface at one end and attached to a magnetic particle at the other end. By generating a magnetic field using external magnets, a force is exerted on the single DNA molecule bound to the magnetic particle. The force applied to the single DNA molecule can be determined from both the applied magnetic force and fluctuations of the magnetic particle position. Thus, by determining the applied force, the force of the action and behavior of protein with the single DNA molecule can be determined by tracking the magnetic particle [64,65]. In addition, magnetic tweezers can generate a rotating magnetic field via a magnet rotation; therefore, they can induce supercoiling of a specified density in the single DNA molecule (Figure 6). For these reasons, magnetic tweezers can be used to analyze the mechanical properties of the various physical forms of DNA, which change in response to DNA-binding protein and enzyme activity [66,67,68,69]. However, tracking the behavior of magnetic beads makes it difficult to analyze the position of protein bound to a single DNA molecule and that of the non-B DNA structures induced by supercoiling.

By using a fluorescence microscope equipped with magnetic tweezers in a microchannel, a single-molecule manipulation system has been developed [70,71]. This system can be used to control the supercoiling density of single DNA molecules. Single DNA molecules are supercoiled by rotating the magnetic field generated by rotating a magnet above the microscope stage. By placing the magnet downstream of the microflow channel, the supercoiled single DNA molecules are stretched, and this can be observed under a microscope. By using magnetic tweezers, the full length of the supercoiled single DNA molecules can be directly observed under a fluorescence microscopic field.

### 2.6. DNA Manipulation by Interface Movement

**Figure 7:**
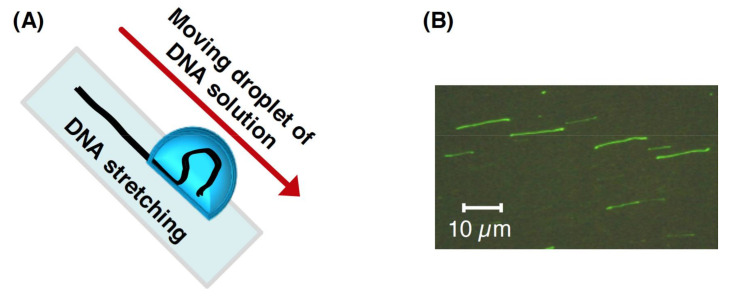
Stretching single DNA molecules using the molecular combing method. (**A**) Conceptual diagram of the stretching manipulation of single DNA molecules by using the moving droplet method. (**B**) Image of the stretched single DNA molecules. Refer to [81] for details.

Around an interface between a liquid phase and a solid phase, or between a liquid phase and a vapor phase, DNA molecules, which are highly hydrophilic materials, tend to maximize the contact area in an aqueous solution. By moving the interfaces between the vapor, liquid, and solid phases, some DNA molecules in the liquid are left behind the interface, which results in the stretching of the DNA molecules [72,73,74]. Based on these properties, the stretching methods for individual DNA molecules have been used in optical mapping. In the molecular combing method, DNA with one end immobilized on a glass surface was stretched by moving interfaces between the vapor, liquid, and solid phases [74,75]. For example, the following molecular combing methods have been developed: (i) the stretching of DNA molecules by sliding another coverslip to a droplet containing DNA molecules on a coverslip [72,76], (ii) the stretching of DNA molecules by the air blowing of a droplet containing DNA molecules on a coverslip [77], (iii) the stretching of DNA molecules by DNA molecule absorption using filter paper [78], and (vi) the dipping of a coverslip into a solution containing DNA molecules and then the stretching of the DNA molecules by lifting up the coverslip (dynamic molecular combing) [79,80]. In another attractive approach, droplets containing DNA molecules are slid along a slope of a coverslip modified using 3-aminopropyltriethoxysilane (APTES) (Figure 7A) [81,82]. This method is based on the adsorption of the DNA molecules with a negative charge of the phosphate backbone on the amino silane-treated glass surface. The sliding of the droplets enables the movement of the interface between the vapor, liquid, and solid phases, which results in the stretching of the single DNA molecules (Figure 7B). In this method, the sliding speed of the droplet can be controlled by the slope angle of the slide glass. Moreover, the sample can be recovered using Parafilm placed under the slide glass, which reduces the amount of droplet that needs to be used. Using molecular combing methods, the sequence-specific positions of stretched DNA molecules can be utilized as optical mapping, facilitating the application of genome-wide analysis (see Section 3.2).

### 2.7. DNA Origami and Control of DNA Folding

DNA, the genetic information carrier, has been playing wide roles as an attractive material for the construction of nanoscale structures [83,84,85]. Pioneering work by Paul Rothemund has significantly expanded the potential for designing and building arbitrarily shaped, complex, two- and three-dimensional nanostructures with DNA (DNA origami) [86]. DNA origami is achieved by controlling self-folding based on the molecular DNA sequence. In the DNA-origami technique, a long single-stranded DNA (scaffold DNA) is hybridized with hundreds of short synthetic DNA oligonucleotides around 30 nt in length (staple strands). Each staple strand is designed to be complementary in sequence to different parts of the scaffold DNA [87]. For this reason, the staple strands are hybridized to distant parts of the scaffold DNA (e.g., crossover points), resulting in inducing the mechanical folding of the scaffold DNA into a designed nanostructure. The development of DNA-origami research has dramatically improved the complexity of DNA designs for building 2D and 3D nanostructures. Thus, DNA-origami-frame-based nanotechnology has been used in various research fields including DNA–protein interactions [88,89], the nanofabrication of nanoelectronics and nanophotonics devices [90,91,92], biosensors [93,94], nanopores [95,96], and drug-delivery systems [97,98].

In several previous studies, the dynamic behavior of and interaction between DNA and protein have been directly observed by high-speed AFM (see Section 3.7) [99,100]. However, the conformation of DNA molecules in an aqueous solution flexibly fluctuates due to Brownian motion, as described in Section 1.2. Therefore, it is difficult to obtain the dynamic behavior of and interaction between protein and relaxed DNA. To manipulate single DNA molecules at the nanolevel, a DNA-origami structure, called a DNA-origami frame, was designed and built by the group of Paul Rothemund et al. The DNA origami frame can accommodate two different DNA fragments in the cavity, resulting in incorporating any modified DNA and RNA strands [101]. The physical form such as tensions, rotations, and the torsion of incorporated DNA can be manipulated by inducing two different DNA strands into four connection sites in the DNA-origami frame [102]. In addition, the orientations of two dsDNA strands or four ssDNA strands can be manipulated via the DNA-origami frame, resulting in manipulating the arrangement of the incorporated DNA strands. The four different ssDNA strands, three-way branched strand, and four-way branched strands formed in the DNA-origami frame have been used for the formation of the G-quadruplex [102,103,104], a substrate of proteins acting on DNA [88,89,105].

### 2.8. Nanopores and DNA Manipulation

Nanopore technology has been used as a platform to identify the structure and sequence of single-molecule DNA or RNA [106]. *Cis* and *trans* electrode chambers are separated from a thin membrane with a nanopore that connects the conductive solution and the target biomolecules [107]. Upon applying a voltage across the membrane with a nanopore, electrolyte ions flow through the nanopore, resulting in highly sensitively detecting the formed pore current. By excluding the electrolyte ions from the pore current, a translocating biomolecule can be detected by monitoring the changes in the current. Nanopore systems are divided into the two types of solid-state nanopores and protein nanopores [108]. Solid-state nanopores are functional over wider ranges of temperatures, voltages, and solvent conditions and can be tuned in diameter with subnanometer precision, resulting in advantaging solid-state nanopores as compared to biological/protein nanopores [109]. However, nanopores have the two major issues of low spatial and low temporal resolutions. In the obstacle of the low spatial, dozens of bases can pass solid-state nanopores at a time because of the thickness of solid based materials, resulting in limitation of the distinguish the four kinds of base [109]. In the obstacle of the temporal resolution, DNA translocation is too fast under a strong electrical force, which is required in drawing DNA into the nanopore, resulting in the limitation of the acquisition of valid data points. To solve this problem, the speed of DNA translocation through nanopores has been controlled by DNA manipulation techniques such as optical tweezers [110] and magnetic tweezers [111]. The force for drawing a single DNA molecule inside a nanopore was measured by combining optical tweezers with ionic-current detection. A force opposing the force for drawing the single DNA molecule inside the pore can be exerted by the optical tweezers. Thus, DNA tension induced by optical tweezers reduced the speed of DNA translocation inside a nanopore [110]. The speed of the translocating DNA inside a nanopore can be controlled by the balance between the electrical force for drawing a single DNA molecule into a pore and the gradient of the magnetic field on the magnetic bead exerted by magnetic tweezers. Using magnetic tweezers, thus, the DNA tethered to the magnetic beads can be slowly translocated into a nanopore [111]. AFM can also be applied to controlling DNA translocation in a pore. DNA tethered to the tip of cantilever can be manipulated through an AFM tip. The tension and electrical forces for drawing a single DNA molecule can be measured through manipulating DNA inside a nanopore. These measurements revealed the kinetics for translocating DNA inside nanopores, indicating the appropriate forces necessary for drawing a single DNA molecule [112].

As a third type of nanopore that is completely different from the protein nanopores and the solid-state nanopores, a combination of nanopores and DNA origami has been developed. Through the manipulation of the mechanical folding of DNA origami, DNA-origami nanoplates and origami nanopore blockages are designed with accurate shapes and sizes. The speeds of translocation in a nanopore can be controlled by controlling the interactions between biomolecules and DNA scaffolds [113,114,115]. As an application example of DNA sequencing, the ionic conductivity was monitored using nanopores on graphene docked with layered DNA origami. The four types of DNA bases were distinguished according to the blockade current, according to the specific interactions between the DNA-origami plate layers and the different DNA bases. Thus, the translocation speeds and detection of DNA in a nanopore can be controlled by the interactions between target biomolecules and the DNA scaffold [116,117]. By introducing various modifications (e.g., sensing elements, such as DNA, fluorophores, and FRET (see Section 3.1.3), and moving parts) at specific sites of DNA origami, DNA-origami nanopores have provided new insight as a powerful tool for single-molecule detection [116,117]. In the near future, DNA-origami nanopores may be achieved in the development of variable and dynamic nanopore structures.

## 3. Single-Molecule Imaging for Biological Processes

By manipulating DNA, the randomly coiled state of a single DNA molecule can be controlled toward stretched and supercoiled states. Unlike in the randomly coiled state, the position of a protein bound to single DNA molecules under the stretched state can be determined from the length information of the DNA molecules. Thus, single-molecule techniques combining DNA manipulation and single-molecule imaging have successfully analyzed the dynamic interaction between DNA and protein, thus providing a new insight into the elementary processes of DNA replication, repair, recombination, and others.

Here, we provide a summary of the biological processes on the basis of the combination of both DNA manipulation and single-molecule imaging as follows: (1) the fluorescence labeling of biomolecules for single-molecule imaging, (2) optical mapping on single DNA molecules, (3) single-molecule imaging for ssDNA molecules by fluorescent ssDNA-binding protein, (4) single-molecule imaging for DNA digestion by exonuclease, (5) single-molecule imaging for DNA synthesis by DNA polymerase, (6) single-molecule imaging for supercoiled DNA and DNA secondary structures, (7) single-molecule imaging for DNA origami, (8) single-molecule imaging for the initiation of DNA replication, and (9) single-molecule imaging based on zero-mode waveguides.

### 3.1. Fluorescence Labeling of Biomolecules for Single-Molecule Imaging

#### 3.1.1. Fluorescence Staining of Double-Stranded DNA

Fluorescence microscopy has allowed the analysis of the dynamic behavior of single DNA molecules in an aqueous solution. However, nonlabeled DNA, which has no fluorescent properties, is not captured by fluorescence microscopy techniques. For this reason, DNA must be labeled with a fluorescent compound. To date, a large variety of fluorescent compounds have been developed as DNA-labeling dyes. These fluorescent compounds can be categorized into three types: (i) those binding to the minor/major grooves of double-stranded DNA (dsDNA), (ii) those intercalating between the base pairs of the dsDNA, and (iii) those doing both [118,119,120,121,122]. Among them, fluorescent dyes, such as YOYO-1 and SYTOX Orange, which are intercalated between the base pairs of the dsDNA, have been utilized for the single-molecule imaging of DNA molecules. These intercalating dyes, which are cyanine dyes, are largely nonfluorescent in solution and interact with dsDNA with high affinity [123,124,125,126], resulting in a 1000-fold increase in fluorescence intensity. Thus, single DNA molecules are easily detected with increases in the signal-to-background ratio. These fluorescent dyes are essential tools for single-molecule imaging, but several studies have revealed that parameters such as the contour length and the persistence length of DNA were significantly changed by the binding of fluorescent dyes, irrespective of their DNA-binding mechanisms [127,128]. These parameters are strongly dependent on concentration and fluorescence dye type; therefore, these should be considered in single-molecule experiments.

#### 3.1.2. Fluorescence Labeling of DNA-Binding Proteins

Analysis of the interactions between DNA and proteins has been an important component of single-molecule approaches for investigating elementary biological processes such as DNA replication, repair, and recombination. Target proteins can be easily fused with a fluorescent protein to the N- or C-terminal of a target protein. To achieve this goal, it is necessary to build an expression vector in which a fluorescent protein gene is inserted either upstream or downstream of the target protein gene. Using a protein expression system from bacterial, insect, and mammalian cultivated cells, the target fusion protein is prepared by extracting it from the cells, followed by purification. However, because the molecular weights of fluorescent proteins are larger than those of peptide tags (e.g., EGFP; 27 kDa), the fusion protein may form an inclusion body due to inappropriate protein folding. In addition, the fusion often impairs the activity of the target protein. To prepare a fusion protein, thus, it is necessary to clearly understand the function and structure of the target proteins. 

Because the labeling of DNA-binding proteins with fluorescent protein is quite effective for investigating DNA–protein interactions, fluorescent-labeling technologies for DNA-binding proteins have also been developed [129,130]. The fluorescent-labeling approaches were achieved by fusing self-labeling protein tags (e.g., SNAP-tag, CLIP-tag, or HALO-tag) to a target protein. The fusion protein can be labeled by attaching various colored fluorophores. The bright fluorophores are designed to bind to the self-labeling protein tags. The use of the self-labeling protein tags allows avoiding the steric hindrance effect caused by the fusion of fluorescent protein, resulting in a far brighter and more photostable fluorophore.

In the above methods using a fusion protein for fluorescence protein preparation, the fluorescence activity may be insufficient for single-molecule imaging, as the number ratio of the molecules of the fluorescence protein to those of the target protein is only 1:1. To solve this problem, the methods have been developed such that the surface of the proteins is modified with fluorescence chemicals while protecting the active sites of the proteins [131]. In particular, DNA-binding proteins catalyze by specifically binding to DNA. This means that the active sites of DNA-binding proteins are protected by the binding of DNA during DNA–protein interaction. By modifying with fluorescent compounds or using a crosslinking agent, thus, it is possible to prepare a highly fluorescently labeled target protein without losing the activity [131,132]. Using this method, a restriction enzyme was labeled with an amine-reactive fluorescent moiety, facilitating the optical mapping of specific positions of DNA sequences (see Section 3.2).

#### 3.1.3. Single-Molecule Imaging by Single-Molecule FRET

Förster resonance energy transfer (FRET) has been used as a powerful tool to quantify putative interactions between neighboring biomolecules, such as protein–protein interactions, protein–DNA interactions, and also protein conformational changes [133,134]. For monitoring the interaction between two biomolecules, one of the biomolecules is fluorescently labeled as a donor fluorophore, and the other, as an acceptor one. When the donor and the acceptor biomolecules are separated by less than approximately 10 nm, the excitation energy of the donor, which reaches an excited electronic state, is transferred to the acceptor through the electronic resonance of molecular orbitals [133,134]. By nonradiative energy transfer between a donor and an acceptor molecule, thus, the FRET is able to identify interactions between the labeled complexes. 

Single-molecule FRET (smFRET) facilitates measurement for the dynamic interactions and behavior between a fluorescently labeled donor and an acceptor, such as protein–protein and protein–DNA [135,136,137,138,139] interactions. In addition, by labeling with a donor and an acceptor at different sites on the same single molecule, the conformation changes within a single molecule are allowed to be observed [140,141]. However, it is necessary to understand the overall structure of the molecule or complex of molecules and the site-specific attachment of fluorophores and color of the fluorophores. 

SmFRET has been applied to the analysis of the DNA-unwinding mechanisms of various DNA helicases acting on single DNA molecules. With one smFRET-based approach, the DNA-unwinding mechanisms of Mcm2–7 helicase in eukaryotic DNA replication have been analyzed [142,143,144]. Based on the closed Mcm2–7 ring structure, the Mcm2 and Mcm5 were fluorescently labeled with a donor and an acceptor, respectively [144]. This is because ATP binding at the Mcm2–Mcm5 interface is purported to close the Mcm2–7 ring by cryo-EM and negative-stain EM studies. For this reason, the opening and closing of *S. cerevisiae* ring-shaped Mcm2–7 DNA helicases were monitored during the recruitment of the pre-replicative complex (pre-RC) to the origin of replication. The application of smFRET provided new insights into the mechanism of eukaryotic DNA replication. Through another smFRET-based approach, the DNA-unwinding mechanisms of the WRN helicase of Werner syndrome (WRN), which is caused by mutations in the WRN gene encoding WRN helicase, have been analyzed [145]. To evaluate the DNA-unwinding properties of WRN helicase, the substrates of different DNA structures, including forked DNA, overhanging DNA, and G-quadruplex-containing DNA, were fluorescently labeled as a donor and an acceptor, respectively. The DNA unwinding of WRN helicase was not caused by complete dissociation from and rebinding to substrates or by strand switching, but by the reciprocating of WRN moving along the same ssDNA substrates. The repetitive movements were shown to behave differently for each DNA substrate, such as forked DNA, 3′/5′-overhanging DNA, and G-quadruplex-containing DNA. These functions of WRN helicase assist the access of other enzymes acting during biological processes, such as DNA replication, repair, and recombination, resulting in it being helpful for the inheritance and maintenance of genome stability.

### 3.2. Optical Mapping on Single DNA Molecules

A restriction enzyme binds and cleaves double-stranded DNA (dsDNA) molecules at specific restriction sites. This characteristic of the restriction enzyme can be employed for the optical mapping of restriction sites on single DNA molecules [146,147,148,149]. In the optical mapping of a DNA-binding protein, the fluorescent labeling of the target protein is necessary. Because fluorescently labeled reagents often attack the active sites of a protein and impair the protein functions, the active sites should be protected [150,151]. In the absence of magnesium ions, most restriction enzymes bind to the DNA sites of recognition sequences but do not progress to cleavage reactions. Therefore, the active sites of the restriction enzymes are protected by binding the enzyme to the DNA molecules in a buffer solution without magnesium ions. Then, the enzyme bound to the DNA molecules is stained with an amine-reactive fluorescent dye. As a result, the catalytic site of the enzyme is protected from modification by the amine-reactive fluorescent dye. Finally, the fluorescently labeled restriction enzyme can be released by cleaving the bound DNA molecule via the addition of magnesium ions to the solution. In single-molecule imaging, fluorescently labeled *Eco*RI enzyme was observed at five specific recognition sites on stretched lambda DNA molecules without magnesium ions [150]. The positions of the *Eco*RI binding and cutting sites on the lambda DNA molecules are in line with the restriction map of the nucleotide sequence [147,150]. Through optical mapping, the specific binding sites of the DNA-binding protein in the single DNA molecules can be determined from the length information of the DNA molecules. 

As another single-molecule approach for optical mapping, DNA methyltransferases (MTases) have been used [152,153,154]. In DNA methylation, DNA MTases catalyze the transfers of methyl groups from the ubiquitous cofactor S-adenosyl-L-methionine (AdoMet) to specific positions of DNA sequences [155]. By using synthetic cofactor analogues, specific positions of DNA sequences can be labeled via engineered DNA MTases [156]. The optical mapping approaches for DNA sequences by the conjunction with synthetic cofactor analogues and DNA MTases are divided into the two types of the methyltransferase-directed transfer of activated groups (mTAG) [157,158] and methyltransferase-directed labeling [159]. The fluorescent labeling of DNA sequences using mTAG is a simple, two-step procedure [157,158]. In the first step, the specific positions of DNA sequences were aminated by DNA MTases and synthetic cofactor analogues. In the second step, the aminated DNA was fluorescently labeled by an amine-reactive fluorophore, resulting in optical mapping from single DNA molecules immobilized on a surface. In a methyltransferase-directed labeling approach [159], the covalent attachment of a fluorophore to a specific DNA sequence was catalyzed by the DNA MTase M.TaqI, from *Thermus aquaticus*. The fluorescent labeling of MTase on DNA was achieved in a single step by using a synthetic cofactor containing a fluorophore at the transfer position. The labeling positions of the stretched DNA molecules were analyzed from the fluorescence signals, resulting in the accurate genotyping of the genomes of λ and T7 bacteriophages. These methods demonstrated that the analysis of specific positions of genomic DNA can be advantageous for genome-wide analysis [160,161,162].

### 3.3. Single-Molecule Imaging for ssDNA Molecules by Fluorescent ssDNA-Binding Protein

**Figure 8:**
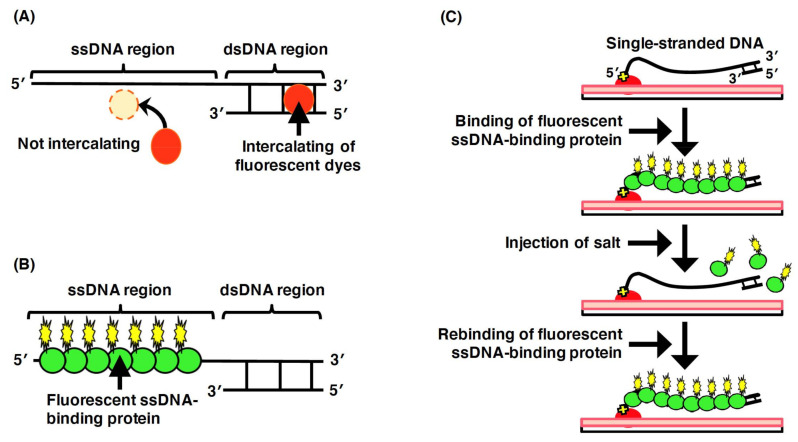
Labeling of the single-stranded regions of single DNA molecules with fluorescent ssDNA-binding proteins. (**A**) Staining the double-stranded region of a DNA molecule with an intercalating dye. The single-stranded region is not stained with an intercalating dye. (**B**) Labeling of the single-stranded regions of DNA molecules with fluorescent ssDNA-binding proteins. (**C**) Schematic illustration of the dynamic behavior of the single-stranded DNA molecules labeled with fluorescent ssDNA-binding protein. One end of ssDNA molecule was specifically immobilized on modified glass surface, and then, fluorescent RPA molecules were injected into the flow cell, resulting in the binding of fluorescent RPA to the ssDNA molecules. The dynamic behavior of the physical form of ssDNA molecule was monitored with and without fluid flow. When salt was added at more than 200 mM, the fluorescent RPA molecules were released from the ssDNA molecule. The ssDNA was restained with the fluorescent RPA after removing the salt. Refer to [166] for details.

To directly observe DNA molecules under a fluorescence microscopic field, the biomolecules need to be labeled using a fluorescent chemical compound (see Section 3.1.1). Intercalating dyes (e.g., YOYO-1 and SYTOX Orange), which are intercalated between the base pairs of dsDNA, can be utilized to directly observe the double-stranded regions of DNA molecules (Figure 8A). However, fluorescent intercalating dyes are not applicable for the direct observation of the single-stranded regions of DNA molecules. However, in the elementary processes of DNA replication, repair, and recombination, single-stranded regions of DNA molecules are generated by proteins acting on DNA, such as DNA helicase and DNA exonuclease. Thus, to analyze the elementary biological processes, the single-stranded regions of the DNA molecules need to be directly observed.

To enable the direct observation of the single-stranded regions of DNA molecules, ssDNA-binding proteins are used (Figure 8B) [82,163]. In particular, eukaryotic replication protein A (RPA), which is composed of the heterotrimeric subunits of 70, 32, and 14 kDa, is an ssDNA-binding protein that binds to the phosphate group of ssDNA [164,165]. Using a fusion protein between the ssDNA-binding domain of RPA and a fluorescent protein (see Section 3.1.2), the single-stranded region of a single DNA molecule has been directly observed under a fluorescence microscopic field [82,163].

In single-molecule imaging using a microchannel device, ssDNA molecules fluorescently labeled with ssDNA-binding proteins are stretched with the fluid flow, whereas are they randomly coiled without the fluid flow [166]. As an effect of the salt, the fluorescent ssDNA-binding protein, RPA–YFP molecules bound to the ssDNA molecules are released by a high concentration of sodium chloride, and then, the RPA–YFP molecules are rebound to the ssDNA molecules under a salt-free condition [166] (Figure 8C). In the presence of free DNA-binding protein (e.g., RPA, SSB, and Rad51) in the solution, the RPA molecules dissociate from the single-stranded regions of DNA molecules, which results in a rapid exchange between the free and bound states of RPA molecules [167]. Thus, single-molecule imaging has revealed that the ssDNA-binding affinity of RPA is not strong but that RPA is reversibly bound to ssDNA. These results indicate that RPA protects ssDNA. However, RPA is easily displaced from ssDNA; therefore, other proteins acting on DNA are allowed to access the ssDNA. Therefore, fluorescent ssDNA-binding protein is highly effective for the single-molecule imaging of the elementary process of DNA replication, repair, and recombination and others.

### 3.4. Single-Molecule Imaging for DNA Digestion by Exonuclease

Exonucleases with DNA digestion activities starting from either the 3′ or the 5′ end play significant roles in the maintenance of genomic stability, such as DNA replication, repair, and recombination [168,169]. In particular, the exonuclease activity exists in an independent protein and protein combined with other enzymatic activities. For instance, DNA polymerase is known to exhibit both DNA synthesis activity and exonuclease activity. The exonuclease activity functions to rectify the incorrect pairing of bases during DNA synthesis [170]. To analyze the dynamic DNA digestion by exonuclease, it is significantly effective to directly observe it at the single-molecule level [56,171,172,173,174]. Thus, by combining DNA manipulation and single-molecule imaging, the elementary process of DNA digestion by λ exonuclease [56,171], exonuclease III [172,173], and T7 exonuclease [174] has been successfully captured.

**Figure 9:**
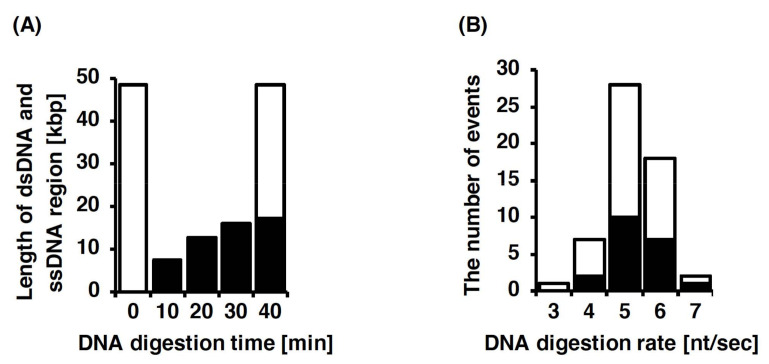
Analysis of the single-molecule imaging of the exonuclease activity on single DNA molecules. (**A**) Time course for the length of the double-stranded region and single-stranded region of a single DNA molecule during DNA digestion by T7 exonuclease. White bars indicate the length of the double-stranded region in single DNA molecule stained with SYTOX Orange. Black bars are the length of the single-stranded region digested by T7 exonuclease stained with fluorescently labeled ssDNA-binding protein. Sum of the lengths of single- and double-strand regions agreed with that of the undigested DNA at 40 min of DNA digestion time. (**B**) Histogram of the DNA digestion rates for T7 exonuclease. White bars and black bars indicate the results determined from single-molecule imaging of DNA digestion under pulse-chase conditions and under continuous conditions with T7 exonuclease. Refer to [174] for details.

In the direct observation of DNA digestion by T7 exonuclease, the activity of T7 exonuclease was analyzed on the basis of the length of the single DNA molecule stretched by fluid flow [174]. The single-stranded and double-stranded regions of the single DNA molecule were stained with two different fluorescent dyes. During the observation, the double-stranded region was monotonously shortened by the DNA digestion of T7 exonuclease, whereas the single-stranded region, as a product, was monotonously elongated from the free end (Figure 9A). Furthermore, the DNA digestion of T7 exonuclease was directly observed under a pulse-chase condition and continuous buffer flow conditions with T7 exonuclease. In the pulse-chase condition, T7 exonuclease was only supplied when the reaction started. This result indicates that the single DNA molecules were gradually digested by the following process, the cycling of the binding, digestion, and dissociation of T7 exonuclease. The average rate of DNA digestion was estimated to be 5.3 ± 0.6 bases/s, with a processivity of 5072 ± 1554 bases (Figure 9B).

### 3.5. Single-Molecule Imaging for DNA Synthesis by DNA Polymerase

DNA polymerase, which catalyzes the synthesis of DNA strands with complementary nucleotide sequences to ssDNA as a template, plays a significant role in DNA replication and repair. Six types of DNA polymerase have been discovered in prokaryotes (five types in *Escherichia coli*) [175], and 15 types, in eukaryotes [176], allowing it to perform various functions, such as DNA replication and base-excision repair. Based on the functional analysis of DNA polymerase, DNA synthesis models, such as leading- and lagging-strand synthesis and translation DNA synthesis (TLS), have been proposed. However, in these models, the following processes of DNA synthesis have not been sufficiently elucidated: (i) the dynamic behavior, such as the binding, synthesis, dissociation, and pauses of DNA polymerase with template DNA; (ii) the replacement process between DNA polymerases during DNA synthesis; and (iii) the processivity and speed of DNA polymerase. To demonstrate the process of DNA synthesis, it is significantly effective to directly observe it at the single-molecule level. This is because single-molecule imaging has provided new insights into the elementary process of DNA synthesis by DNA polymerase [177,178,179,180].

**Figure 10:**
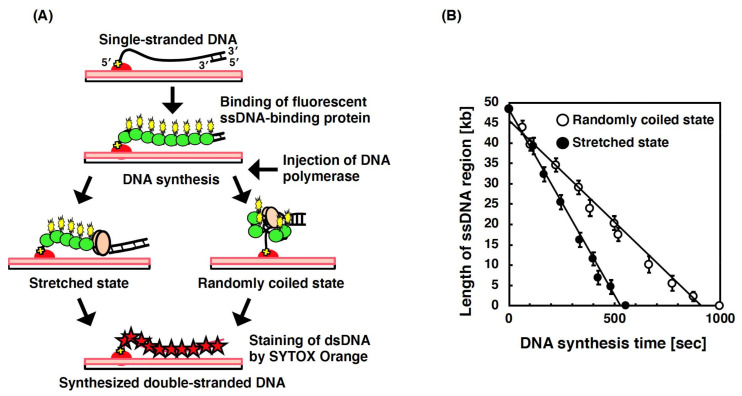
Analysis of the single-molecule imaging of the activity of DNA polymerase on single DNA molecules. (**A**) Schematic illustration of the DNA synthesis by DNA polymerase at a single-molecule level. One end of template ssDNA for DNA synthesis was specifically immobilized on a modified glass surface, and then, fluorescently labeled RPA molecules were injected into the flow cell, resulting in the binding of fluorescently labeled RPA to the ssDNA molecules. During DNA synthesis by DNA polymerase, the DNA tensions under the randomly coiled state and stretched state were controlled in the presence or absence of fluid flow. After the DNA synthesis, the dsDNA synthesized from ssDNA was stained by SYTOX Orange, allowing visualizing under a fluorescence microscopic field. (**B**) Time course for the length of the single-stranded regions of the single DNA molecules under the relaxed and stretched states during DNA synthesis by Klenow fragment (3′–5′ exonuclease). Closed circles and open circles indicate the length of the single-stranded region on stretched ssDNA and that on relaxed ssDNA, respectively. Refer to [183] for details.

In the single-molecule technique for DNA synthesis by DNA polymerase, DNA synthesis was directly observed using template ssDNA labeled by fluorescent ssDNA-binding protein (Figure 10A). An *E. coli* DNA polymerase I mutant, namely, Klenow fragment (3′–5′ exonuclease minus), lacks the exonuclease activities for both directions (5′–3′ and 3′–5′) and has only the polymerase activity of DNA polymerase I [181,182]. This means that the digestion of the template ssDNA by the exonuclease activity of DNA polymerase I was eliminated. Therefore, only the DNA synthetic reaction was directly observed in this single-molecule experiment [183]. Figure 10A shows the scheme of DNA synthesis by DNA polymerase at the single-molecule level. The DNA tensions under the randomly coiled state and stretched state were controlled in the presence or absence of fluid flow. The analyses were conducted on the basis of the length of the stretched single DNA molecule. Under the randomly coiled state, the activity of DNA polymerase was measured via the temporary stretching of the single DNA molecule. Conversely, under the stretched state, the activity of DNA polymerase was measured via the continuous stretching of the single DNA molecule. As a result, the synthesis rate of DNA polymerase for the stretched DNA was approximately 75% higher than that for the randomly coiled DNA molecules (91 nt/s vs. 52 nt/s) (Figure 10B). These results indicate that the physical form of DNA significantly affects the activity of DNA polymerase.

### 3.6. Single-Molecule Imaging for Supercoiled DNA and DNA Secondary Structures

It has been strongly suggested that the physical form of DNA affects the activity of enzymes acting on DNA [51,53,173,183,184]. This indicates that the physical forms of DNA, such as supercoiling and supercoiling-induced non-B-DNA structures, may play significant roles in the regulation of DNA replication, repair, recombination, and others. To induce supercoiling of a specified density in a single DNA molecule, magnetic tweezers have been used (see Section 2.5). The superhelicity of single DNA molecules has been controlled using magnetic tweezers under a fluorescence microscopic field [70]. Single-molecule imaging has revealed the following: (i) plectonemes were induced by supercoiling; (ii) plectonemes were moved along a single DNA molecule by diffusion; (iii) new plectonemes were induced at a distant position via a fast hopping process. It has been suggested that the dynamic behavior of plectonemes may enhance protein binding and gene expression as follows [185]. In single-molecule imaging, the plectonemes on single DNA molecules were simply observed by the intercalating of SYTOX Orange between base pairs of DNA. It was shown that plectonemes were induced upstream of promoters in several prokaryotic genomes. 

It has been proposed that DNA looping with DNA supercoiling plays critical roles in the spatial organization of chromosomes. Structural maintenance of chromosome (SMC) protein complexes such as condensin and cohesion play key roles in restructuring genomes during the cell cycle [186,187]. The formation and processive extension of DNA loops by yeast condensin on single DNA molecules was directly observed via single-molecule imaging [188]. The elegant single-molecule experiment demonstrated that by the action of a condensin complex, tens of kilobase pairs of DNA were extruded as a loop structure, at a force-dependent speed of up to 1500 base pairs per second. Single-molecule imaging has shown that the induction of DNA loop extrusion by SMC complexes may be the key principle for the organization of genome architecture.

In single-molecule imaging for DNA secondary structures induced by supercoiling, local structure denaturation of single DNA molecules was frequently induced under a high negative supercoiling density but was almost not induced under a low negative supercoiling density [71]. Local structure denaturation was often observed at the initiation region for DNA replication on single DNA molecules under a high supercoiling density. These results indicate that negative supercoiling enhances the initiation of DNA replication.

### 3.7. Single-Molecule Imaging for DNA Origami

As described in Section 2.7, DNA-origami-based nanotechnology has been developed for the construction of various custom-made nanostructures. To evaluate the nanostructures of DNA origami, high-resolution visualization is necessary. To date, the nanostructures of DNA origami have been imaged by AFM and/or EM, which is commonly used to directly acquire high-resolution images of nonlabeled biomolecules under physiological conditions [189]. However, the speed performance of AFM imaging was significantly too low to monitor dynamic interactions between DNA and protein. To overcome the limitation, the development of high-speed AFM (HS-AFM) was started around 1993. By extensive improvements of AFM instruments, such as in the cantilevers, detectors, and scanners, HS-AFM has now materialized, resulting in capturing images of biological molecules within 100 ms or less [190,191,192]. HS-AFM imaging has captured a wide range of dynamic events in biomolecular processes [190,191,192,193,194]. Combining the DNA-origami frame (see Section 2.7) and HS-AFM, the dynamic behavior of biomolecular processes, such as enzymatic reactions [88,89,101,195] and DNA structural changes [103,104,196], has been directly observed at a nanolevel. For example, the conformational changes of the G-quadruplex induced in the DNA-origami frame were visualized in real time with HS-AFM [195]. The G-quadruple was formed in the presence of salt, whereas it was disrupted by the removal of the salt. The DNA-origami frame is suitable for visualizing various conformational changes in DNA secondary structures. As a result of recent research on the dynamic interaction between enzymes and DNA-origami frames, the digestion of DNA-origami nanostructures by endonuclease DNase I was directly observed at a single-structure level by HS-AFM [195]. By analyzing the digestion patterns of DNase I for DNA-origami nanostructures, the design of DNase I-resistant DNA-origami nanostructures was suggested as guidelines. The guidelines play an important role in the design for new DNA-origami nanostructures for specific applications such as drug-delivery systems [189]. 

In fluorescence-based optical microscopes, the dynamic behavior of biomolecules at the nano level such as DNA-origami nanostructures cannot be observed due to the diffraction limit of light (half the wavelength of the emission light). This is because photons emitted from two fluorophores closer than the resolution limit cannot be distinguished under a fluorescence microscopic field. However, the diffraction limit in optical microscopy has been overcome by the development of super-resolution microscopes [197,198]. One of the typical super-resolution techniques, stochastic super-resolution, can resolve the individual fluorophores in time through light-emitting the several fluorophores neighbored at separate times. The super-resolution techniques include single-molecule localization methods (SMLM), such as PALM, STORM, and PAINT. The super-resolution techniques are achieved by isolating emitters and fitting images with the point-spread function to the fluorescence of single molecules [199,200]. Point accumulation for imaging in nanoscale topography (PAINT), which is the one of SMLM, allows capturing several points of stochastic single-molecule fluorescence emitted by molecular adsorption/absorption and photobleaching/desorption by using fast and transient fluorescence dyes. For this reason, instead of the stochastic photoactivation of a permanently bound fluorophore, PAINT depends exclusively on the stochastic/temporary binding of a fluorescent ligand. Using the characteristics of PAINT, DNA-PAINT, which is an extension of PAINT, has been used as a powerful tool in the characterization of DNA-origami nanostructures (e.g., including tetrahedrons, triangular prisms, cubes, pentagonal prisms, and hexagonal prisms) [201,202,203]. In DNA-PAINT imaging, the stochastic blinking of targets is achieved by the transient hybridization of short, dye-labeled DNA oligonucleotides to a complementary target DNA molecule. For this reason, the dye-labeled DNA oligonucleotides unbound to the complementary target DNA strands are freely diffused in solution. Thus, when the dye-labeled DNA oligonucleotides are bound to the complementary target DNA molecule, the acquisition of the image is performed in total internal reflection or oblique illumination, resulting in minimal background. For this reason, DNA-PAINT is significantly suitable for fluorescently visualizing DNA nanostructures, such as the folding of DNA origami and incorporation of DNA strands. Because dye-labeled DNA oligonucleotides, which are parts of origami structure, are constantly replenished from solution, DNA-PAINT resists photobleaching. For this reason, high spatial resolutions are achieved by capturing the maximum number of photons from dye-labeled DNA oligonucleotides bound to a complementary target DNA molecule before unbinding from the complementary target DNA molecule. DNA nanostructures with single binding sites spaced 5 nm apart were observed as the “MPI” and “LMU” logos [204]. Thus, DNA-PAINT has provided new insight into the design analysis of DNA-origami nanostructures [205,206,207,208,209].

### 3.8. Single-Molecule Imaging for the Initiation of DNA Replication

DNA replication is initiated from replication origins [210]. The consensus sequence of replication origin is highly conserved among bacteria and budding yeast; however, it is not completely conserved in some eukaryotes, such as flies, frogs, and mammals [210,211,212,213,214]. However, in bacteria and eukaryotes, the initiation of DNA replication is promoted by the negative supercoiling of DNA [215,216,217,218,219]. It has been suggested that the physical form of DNA plays a significant role in regulating the initiation of DNA replication. The large T antigen (Tag) of Simian virus 40 (SV40) has been utilized as a model of DNA replication in eukaryotic cells [220]. SV40 Tag is crucial for SV40 DNA replication and is the only replication factor encoded by a viral gene. The multifunctional protein SV40 Tag acts as a replication initiator and simultaneously exhibits a DNA helicase activity during DNA replication. Thus, it is assembled at the SV40 replication origin and unwinds duplex DNA [220]. 

The initiation of DNA replication using SV40 Tag has been analyzed via single-molecule imaging [221]. In the single-molecule technique, the unwound DNA on the single DNA molecules was labeled by fluorescent ssDNA-binding protein, whereas the double-stranded regions of the single DNA molecules were labeled by fluorescent intercalating dyes. SV40 Tag assembled on the SV40 origin efficiently unwound DNA as a single hexamer translocated on the ssDNA of the lagging strand in the 3′-to-5′ direction. The translocation of SV40 Tag is the same as that of the Mcm2-7 helicase [222,223,224]. These results indicate that the replicative DNA helicase in eukaryotes unwinds a ssDNA on a lagging strand in the 3′-to-5′ direction. The effect of negative supercoiling on the initiation of DNA replication initiated by SV40 Tag has been analyzed by combining DNA manipulation and single-molecule imaging [225]. The increase in the negative supercoiling density stimulated the DNA unwinding more strongly. Furthermore, negative supercoiling was associated with an increased probability, from the assembly of Tag on the SV40 origin, of DNA unwinding. These results indicate that negative superhelicity facilitates the initiation of DNA replication.

### 3.9. Single-Molecule Imaging Based on Zero-Mode Waveguides (ZMWs)

The diffraction of light limits the optical performance of confocal microscopes, resulting in preventing the detection of a single molecule in a crowded environment [226,227,228]. To solve the diffraction limit, zero-mode waveguides (ZMWs) have been introduced for confining the fluorescent signal at the nanometer scale [229,230]. The confined fluorescent signal can be monitored via ZMWs [229]. ZMWs milled in opaque metallic films supported on a glass surface are nanoapertures in nanoscale cylindrical cavities ~100 nm in diameter and height. A ZMW that is an optical waveguide directs light energy into a well, which is smaller than the wavelength of the illuminating light. This allows ZMWs to generate an evanescently decaying intensity profile that provides a detection volume in the range of atto- to zeptoliters, three orders of magnitude below the diffraction-limited confocal volumes [231]. By enhancing the local fluorescence excitation intensity and modifying the fluorescence decay rate inside the ZMWs, the fluorescence brightness per single molecule close to the bottom of the ZMW is made able to be detected with a high signal [232,233]. Since 2002, ZMWs have been widely used for a large range of biophysical and biochemical applications, including DNA sequencing [234,235], enzymatic reaction monitoring [236,237], studying protein–protein interactions [238,239], nanopore sensing [240,241], and FRET [242,243,244]. In particular, single-molecule sequencing technology is commercialized by Pacific Biosystems as single-molecule real-time (SMRT) DNA sequencing [245,246,247,248]. Single-molecule sequencing uses fluorescent dNTPs phospholinked by four different colored fluorophores. The fluorescent signals generated by the incorporation of each nucleotide with DNA synthesis by DNA polymerase are detected and classified via monitoring in ZMWs. Single-molecule sequencing allows long reading exceeding 10,000 base pairs in length. Over the past decade, the capabilities of single-molecule sequencing technologies have made remarkable progress. However, to make them widely available in medical clinics and research laboratories, key challenges still need to be solved, such as the instrument costs, running costs, handling of the instruments, and data analysis.

## 4. Conclusions

DNA manipulation and single-molecule imaging provide new analytical methods in biotechnology. In this review, the following single-molecule techniques, which are powerful tools for DNA manipulation and single-molecule imaging, have been introduced:

- DNA manipulation by orientation forces, optical tweezers, fluid flow, and magnetic tweezers;
- Nanopores and DNA manipulation;
- Single-molecule imaging for double- and single-stranded DNA;
- The optical mapping of single DNA molecules;
- Single-molecule imaging for biological processes between DNA and protein;
- Single-molecule imaging for supercoiled DNA and DNA secondary structures;
- Single-molecule imaging for DNA origami and DNA sequencing.

These single-molecule techniques are maximally utilized through integration with microfluidic devices. Microdevices used in single-molecule analysis have many advantages in terms of reduced sample volumes and the reaction field to be controlled. In particular, the detection frequencies for the biological events between DNA and protein are significantly improved by the reduced distance of molecular diffusion. The further improvement of DNA manipulation and single-molecule imaging techniques will significantly enhance the dynamic analysis of the behavior of and interaction between biomolecules and provide new insights into the mechanisms of elementary biological processes such as DNA replication, repair, and recombination. A knowledge of DNA replication, repair, recombination, and others at a single-molecule level is helpful for not only predicting behaviors at the cellular level but also understanding the inheritance and maintenance of genome stability and diseases associated with DNA replication, repair, and recombination (e.g., aging and cancer). Thus, these single-molecule studies will play important roles as bridges to close the gaps between the research at the molecular level and the research at the cellular level in the fields of biochemistry and molecular biology.

## Data Availability

Not applicable.
